# A50 SUCCESSFUL HEMOSTASIS WITH THROUGH THE SCOPE CLIPS FOLLOWING OVER THE SCOPE CLIP FAILURE IN DIEULAFOY LESION

**DOI:** 10.1093/jcag/gwae059.050

**Published:** 2025-02-10

**Authors:** S Hendis, M Tomaszewski

**Affiliations:** Universite de Montreal Faculte de Medecine, Montreal, QC, Canada; Universite de Montreal Faculte de Medecine, Montreal, QC, Canada

## Abstract

**Background:**

Dieulafoy lesions account for 1-2% of acute GI bleeding and can be difficult to treat endoscopically.

**Aims:**

We present a case a 59-year-old male who presented with hematemesis, melena, and hemodynamic instability.

**Methods:**

Initial esophago-gastro-duodenoscopies (EGD) did not detect a source of active bleeding. Abdominal CT angiography revealed vascular structure in the stomach’s greater curvature, indicating a Dieulafoy lesion. Embolization via interventional radiology was unsuccessful. A third EGD finally showed the bleeding as a focal ooze localized in the proximal part of the greater curvature of the stomach. We used a padlock over-the-scope clip (OTSC). Despite successful deployment and placement of the OTSC, the bleeding persisted.

**Results:**

Ultimately, hemostasis was achieved using five through-the-scope clips in a zipper fashion to the mound of tissue that had been raised by the over the scope clip. We hypothesize that although the OTSC grasped the Dieulafoy lesion, the feeding vessels were not significantly compressed to achieve hemostasis. This case demonstrates the failure of an OTSC to control bleeding from a Dieulafoy lesion, which was ultimately managed with through-the-scope clips.

**Conclusions:**

This underscores the importance of combining traditional and novel endoscopic techniques to achieve hemostasis.

**Funding Agencies:**

None

